# The effects of Bt Cry1Ie toxin on bacterial diversity in the midgut of *Apis mellifera ligustica* (Hymenoptera: Apidae)

**DOI:** 10.1038/srep24664

**Published:** 2016-04-19

**Authors:** Hui-Ru Jia, Li-Li Geng, Yun-He Li, Qiang Wang, Qing-Yun Diao, Ting Zhou, Ping-Li Dai

**Affiliations:** 1 Ministry Key Laboratory of Pollinating Insect Biology, Institute of Apicultural Research, Chinese Academy of Agricultural Sciences, Beijing 100093, China; 2 State Key Laboratory for Biology of Plant Diseases and Insect Pests, Institute of Plant Protection, Chinese Academy of Agricultural Sciences, Beijing 100193, China

## Abstract

The honey bee has been regarded as a key species in the environmental risk assessment of biotech crops. Here, the potential adverse effects of Cry1Ie toxin on the midgut bacteria of the worker bees (*Apis mellifera ligustica*) were investigated under laboratory conditions. Newly emerged bees were fed with different concentrations of Cry1Ie toxin syrups (20 ng/mL, 200 ng/mL, and 20 μg/mL), pure sugar syrup, and 48 ppb of imidacloprid syrups, then sampled after 15 and 30 d. We characterized the dominant midgut bacteria and compared the composition and structure of the midgut bacterial community in all samples using the Illumina MiSeq platform targeting the V3–V4 regions of 16S rDNA. No significant differences in the diversity of the midgut bacteria were observed between the five treatments. This work was the first to show the effects of Cry1Ie toxin on honey bees, and our study provided a theoretical basis for the biosafety assessment of transgenic Cry1Ie maize.

The global area of genetically modified (GM) crops has continued to increase over the past 19 years. In 2014, the global cultivation area of GM crops exceeded 181.5 million hectares, with a remarkable 100-fold increase in hectarage since commercialization began in 1996. Of the crops grown globally, transgenic maize covered 55.2 million hectares and was the second largest planted crop after soybean^1^. The adoption of biotech crops makes a substantial contribution in increasing crop production and cutting poverty. Simultaneously, the biosafety of transgenic crops has also triggered great concerns from both scientific and public groups. One particular concern is that these crops, including maize, may have harmful effects on non-target insects^2^,^3^.

Honey bees are the most important pollinators in natural ecosystems world-wide and play a significant role in biodiversity maintenance and ecosystem function. Therefore, honey bees have been widely recognized as an indispensable non-target insect in the risk assessment of transgenic crops^4^,^5^. The effects of transgenic crops on honey bees have been thoroughly investigated, with a primary focus on survival^6^,^7^, food consumption^8^,^9^,^10^ learning abilities^11^,^12^,^13^, the development of the hypo pharyngeal gland^14^, and the enzyme activity levels of honey bees^15^ exposed to transgenic plants or their insecticidal proteins.

Accumulating evidence has shown that the guts of honey bees harbour relatively simple and specific microbiota important for nutrition and pathogen defence, and a strict interconnection exists between the intestinal microbiota balance and the health status of honeybees^16^,^17^,^18^,^19^,^20^,^21^. Thus, in recent years, researchers have evaluated the biosafety of transgenic plants by studying the intestinal flora of honeybees^22^. The effects of Bt maize on honeybees and the gut bacterial structures of honeybees have been investigated^23^,^24^.

Previous surveys have shown that transgenic maize with the *cry1Ie* gene conferred a high tolerance to both susceptible and Cry1Ac resistant insects and have the potential to be a new Bt-maize variety in China^25^. However, studies assessing the effects of Bt-*cry1Ie* maize on honey bees are scarce. The intent of the current study was to evaluate the effect of Cry1Ie toxin on the midgut bacterial community of worker bees under laboratory conditions through sequencing technology.

## Results

### Changes in the bacterial 16S rRNA gene abundance

**Figure 1:**
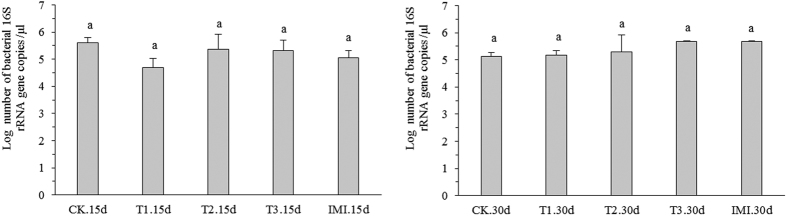
Log-transformed abundance of the 16S rRNA gene across different treatments at 2 sampling times.

As shown in Fig. S1, a standard curve (y = −0.362x + 10.319, R^2^ = 0.995) was established by plotting the threshold cycle (Ct) value against the logarithm of the 16 S rRNA gene initial copy number for each dilution. The 16S rRNA gene copy number in each sample was calculated by a linear regression of the normalized sample Ct values to the standard curve (Table S1). The log-transformed abundance of the copy numbers across different treatments are shown in Fig. 1. For further analyses by one-way ANOVA, no significant differences were observed among different treatments (*P* > 0.05, Table S2). This result showed that Cry1Ah toxin had no effect on the abundance of the bacterial 16S ribosomal RNA (rRNA) gene.

### Basic statistics of V3–V4 16S rRNA gene sequences

In the present study, we characterized the midgut bacterial community of the honeybees via 16S rRNA amplicon Illumina sequencing. After sequencing with the Illumina MiSeq, a quality control procedure, including trimming the barcodes and primers, filtering low-quality reads and chimaeras, on the paired-end reads was performed according to the QIIME. Finally, a total number of 1,388,300 of V3–V4 16S rRNA valid sequences reads were obtained from 30 samples, with an average of 42,070 effective sequences reads for each sample (the minimum of one sample was 31,638 and the maximum was 50,063), and the average length of the effective sequences reads was 420 bp (Table S3). As shown in Fig. S2, the rarefaction curves for all samples have almost approached the saturation plateau, which indicates that the 16S rRNA gene sequence database was very abundant, and the current analysis had adequate depth to capture most microbial diversity information.

### Intestinal bacterial communities of adult honeybees

**Figure 2:**
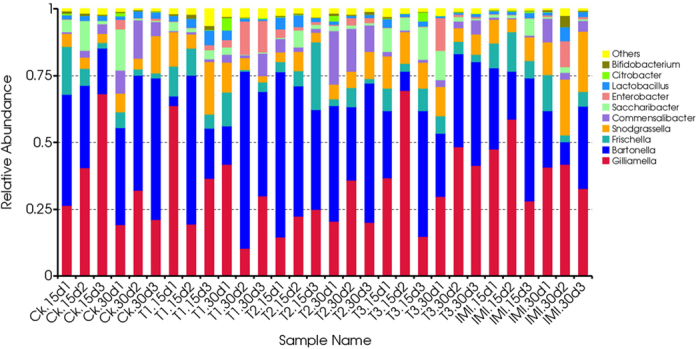
Relative abundance of the dominant bacterial in the midgut communities of *Apis mellifera ligusticaat* at genus level. Each bar represents the relative abundance of each sample. Each colour represents a particular bacterial family. Ck–Pure sugar syrup, T1–20 ng/mL of Cry1Ie toxin syrups, T2–200 ng/mL of Cry1Ie toxin syrups, T3–20 μg/mL of Cry1Ie toxin syrups; IMI–: 48 ppb of imidacloprid syrups.

Subsequently, all valid reads were classified into different taxonomy (phylum, class, order, family, and genera levels) using QIIME. The bacterial diversity and relative abundance of all samples in the different taxonomy are presented in Fig. S3. Six phylogenetic groups, α-, β- and γ-Proteobacteria, Firmicutes, Bacteroidetes and Actinobacteria were identified as the major bacterial taxa of the honeybee bacterial community in all samples. Based on relative abundance, Proteobacteria (mainly γ-proteobacteria) was the most abundant bacterial phylum in the honeybee midgut, with an average relative abundance of over 90%. Firmicutes and Actinobacteria had higher relative abundances compared with other phyla. The taxonomic distribution of each sample at the genus level is shown in Fig. 2. All core bacteria in *A. Mellifera* such as *Gilliamella*, *Frischella*, *Snodgrassella*, *Lactobacillus* and others, have been found in our study. Of all detected genera, *Gilliamella* of the Gamma proteobacteria was the most abundant genus.

### Effects of the Bt Cry1Ie toxin on the midgut bacterial composition of *A. mellifera ligustica*

**Figure 3:**
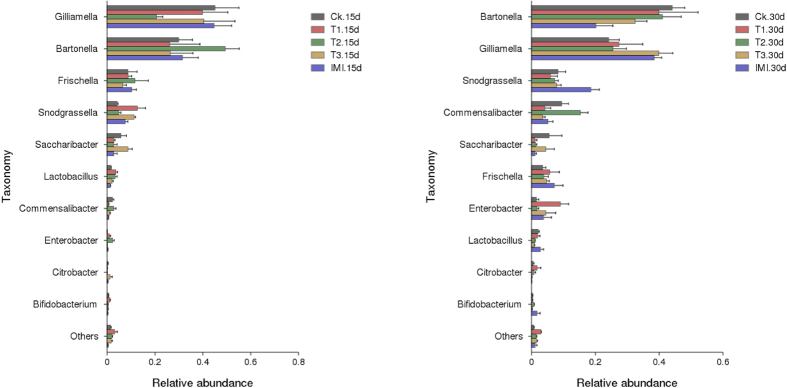
Relative abundance of the dominant midgut bacterial genera in the midgut of *Apis mellifera ligustica* adults across the five treatments at 2 sampling times.

**Figure 4:**
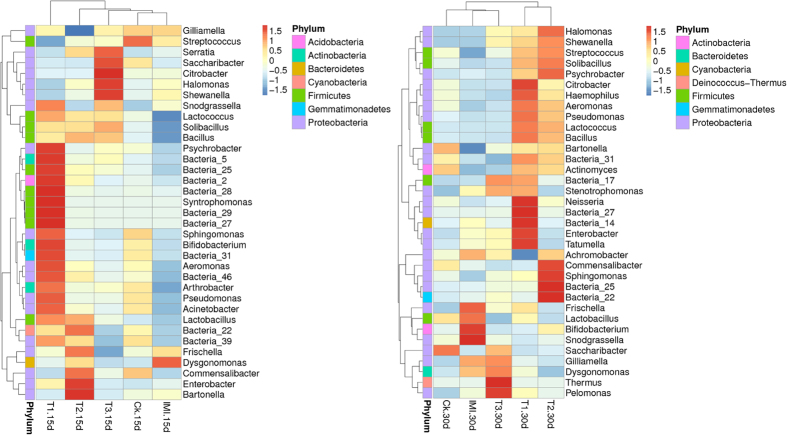
Hierarchically clustered heat map analysis of the highly represented bacterial taxa (at the genus level) found in the midgut of *A. mellifera* workers (relative abundance > 1%) across the five treatments at 2 sampling times. The relative percentages (%) of the bacterial families are indicated by varying colour intensities according to the legend at the top of the figure. The colour key for the Z score indicates correspondence between blue-red colouring and standard deviations from the mean abundance of each bacterial taxon. For those bacterial taxa unable to be assigned into specific known bacterial genera, the higher known taxonomic unit was added: Bacteria_2 (c_Acidobacteria); Bacteria_5 (o_Acidimicrobiales); Bacteria_14 (f_Flammeovirgaceae); Bacteria_17 (c_Cytophagia); Bacteria_22 (p_Cyanobacteria); Bacteria_25 (f_Lachnospiraceae); Bacteria_27 (f_Peptostreptococcaceae); Bacteria_28 (f_Ruminococcaceae); Bacteria_29 (o_Clostridiales); Bacteria_31 (c_Gemmatimonadetes); Bacteria_39 (o_Rickettsiales); Bacteria_46 (f_Enterobacteriaceae).

The five feeding treatments were 20 ng/mL, 200 ng/mL, and 20 μg/mL of Cry1Ie toxin syrups, pure sugar syrup and 48 ppb of imidacloprid syrup. To visualize differences in the bacterial community, the histograms of the dominant midgut bacterial genera were constructed among the different sample groups using the QIIME toolkit (Fig. 3). For statistical analysis, the composition of dominant midgut bacterial genera among different groups was assessed using one-way ANOVA (SPSS. 16.0), and no significant effect was observed in the midgut bacterial community composition of adult honeybees fed on transgenic *cry1Ah*-maize pollen (P > 0.05, Table S4). The shifts of the bacterial community compositions were further corroborated by clear clustering of the dominant bacterial genus corresponding to different treatments in the heat map as shown in Fig. 4.

These results revealed that the midgut bacterial composition in honey bees among the five treatments was not significantly different. Ultimately, the transgenic *cry1Ah*-maize pollen had no significant effect on the midgut bacterial composition.

### Effects of the Bt Cry1Ie toxin on the midgut bacterial structure of *A. mellifera ligustica*

**Figure 5:**
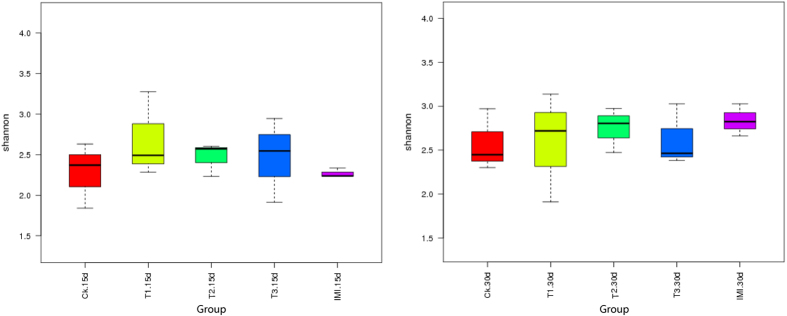
Differences in bacterial community diversity, richness and structures among five sample groups at 2 sampling time, respectively. Box plot of richness estimators of the gut microbiota of the five treatments at 2 sampling times. Each box plot represents each richness estimator. Significant differences are defined at the 95% confidence level.

Alpha diversity was applied to analysing the complexity of the species diversity for a sample. Then, the alpha diversity parameters of each sample, including community richness (ACE, Chao1), diversity indices (Shannon, Simpson), and sequencing depth (Good’s coverage) were determined with QIIME and displayed with R software (Table S5). A box plot of richness estimators was constructed using the QIIME toolkit (Fig. 5), providing a clear visualization of the relationships among the different sample groups. Alpha diversities were further tested by comparing the alpha diversity indexes between groups using one-way ANOVA (SPSS. 16.0), and no significant differences were observed across the five treatments in the same period (*P* > 0.05, Table S6).

To better understand the relationships among the gut microbiota community structures in the honeybees among the treatments, we performed ANOSIM to characterize the differences in the bacterial community compositions across the five treatments. No significant effect was observed in the midgut bacterial community structures of adult honeybees fed transgenic *cry1Ah*-maize pollen (*P* > 0.05, Table S7).

The results indicated that the midgut bacterial community structures in honey bees among the five treatments were not significantly different. Ultimately, the Bt Cry1Ie toxin had no obvious effect on midgut bacterial community structure of honeybees.

## Discussion

Previous studies of the gut bacterial communities of honeybees suggested that the intestinal bacterial community important for nutrition, pathogen defence, and the detoxification of environmental contaminants may be a sensitive indicator for honeybee health^21^,^22^,^26^,^27^,^28^. Multiple lines of evidence have demonstrated that Cry proteins develop their toxicity by forming pores in the gut epithelium of their target insects as a consequence of binding to specific receptors in the epithelial membrane^25^. Therefore, in recent years, researchers have evaluated the biosafety of transgenic plants by studying the intestinal flora of honeybees^23^,^24^. This study aimed to test the impact of transgenic maize with the *cry1Ie* gene on the midgut bacterial community of worker bees under laboratory conditions. However, no apparent effects on midgut bacterial structures and composition were observed. This result was consistent with the previous explorations that showed that Bt-toxin did not significantly affect *A. mellifera* gut bacterial communities^22^,^24^.

More studies on the intestinal microbiota diversity of *A. mellifera* workers have repeatedly shown that honey bees have a co-evolved symbiotic relationship with several characteristic bacterial groups. Six phylogenetic groups, α-, β- and γ-Proteobacteria, Firmicutes, Bacteroidetes and Actinobacteria comprise over 95% of the bacteria in most individuals and were identified as the major bacterial taxa of the honeybee gut bacterial community^17^,^19^,^29^,^30^. This gut community reaches its typical composition during 3–5 days, and then remains stable for the remaining time^31^. Thus, the adult gut appears to have a stable and distinct community. Similar to earlier findings, all dominant bacterial groups have been found in our study, and, in accordance with previous studies, the γ-Proteobacteria was the most common group of bacteria, and Lactobacillus was one of the major diversity genera in honeybee midgut^22^,^24^,^32^.

Many factors influence the microbial diversity and community structure inside bee guts. For example, the microbiome acquisition may be determined by host age and gut morphology. The abundance and community structure of the microbiota changes throughout the *A. mellifera* life cycle and varies among the organs of the adult gut^31^,^33^. Another important element in determining the development and composition of gut communities is the bee health status because pathogen infection can affect gut microbial communities^26^,^34^,^35^. In addition, diet and host species can also perturb gut bacterial community^36^. Thus, to obtain a more reliable result, we needed to control for these external factors, minimizing their contribution to bacterial community variability. In our study, the newly emerged *A. mellifera* workers (less than 12 h old) were randomly sampled from the single apparently healthy apiaries as experimental groups.

A number of methods have been attempted to characterize the honey bee microbiota, including traditional culture-based methods^37^, molecular diagnostics such as denaturing gradient gel electrophoresis (DGGE)^23^,^24^, terminal restriction fragment length polymorphism (T-RFLP)^22^,^32^, and single-strand conformation polymorphism (SSCP)^17^. Herein, we performed Illumina MiSeq sequencing of the 16S rRNA amplicons to investigate the midgut bacterial community of the honeybees. The method is one of the ultra-deep sequencing methods, and these burgeoning technologies have provided novel ways to recognize the microbial community. Beginning in 2003, high-throughput sequencing technologies were used to characterize the honeybee microbiota^16^, and since then, these new approaches have been frequently applied in numerous studies of honeybee microbiota^18^,^20^,^33^. Compared with earlier culture-based methods and molecular diagnostics, non-culture sequencing methods can provide more comprehensive and quantitative information on the bacterial symbionts of honey bees. With these new sequencing technologies, the microbial community can be evaluated with high sensitivity, low cost and short times^38^,^39^.

Although the effects of Bt-maize on honeybees have been extensively conducted recently^6^,^7^,^12^,^13^, a comprehensive investigation of the impact of Bt-maize variety with *cry1Ie* gene on honeybees has been lacking. Our present study established a foundation for the comprehensive understanding of the effects of Bt-maize on honeybees and provided a theoretical basis for the biosafety assessment of biotech crops. We concluded that transgenic *cry1Ie* maize pollen had no significant direct adverse effects of on the midgut bacterial community composition and structure of honeybees (*A. mellifera ligustica*). However, the evaluation of the side effects of Cry1Ie corn pollen on honeybees was merely investigated under laboratory conditions, and the effects under field conditions will require further investigation in the future.

## Methods

### Insect and Cry1Ie toxin preparation

*Apis mellifera* stocks were sampled from the apparently healthy apiaries of the Department of Bee Protection and Biological Safety at the Institute of Apicultural Research, Chinese Academy of Agricultural Sciences, Beijing, China on April 22, 2015. Brood frames were placed in an incubator (33 ± 1 °C, 60 ± 10% relative humidity, darkness) after the cells were capped, and the newly emerged worker bees (less than 12 h old) were randomly assigned to wooden cages (dimensions of 10 cm × 7 cm × 8 cm) with mesh on two sides.

Cry1Ie toxin was provided by the State Key Laboratory for Biology of Plant Diseases and Insect Pests, the Institute of Plant Protection, Chinese Academy of Agricultural Sciences (Beijing, China). To identify whether the concentration of Cry1Ie toxin influenced the intestinal bacterial community of the honeybees, three concentrations (20 ng/mL, 200 ng/mL, and 20 μg/mL) of Cry1Ie toxin were used in the experiment. Cry1Ie toxin was mixed thoroughly into sugar syrup (60% w/v sucrose solution) to obtain the desired concentrations.

### Laboratory feeding of honeybees and experiment design

Newly emerged worker bees (less than 12 h old) were used in this study. Each cage contained 30 bees and was kept in a dark incubator at 29 ± 1 °C with 65% ± 5% relative humidity. Five treatments were designed to examine the effects of the Cry1Ie toxin on the intestinal bacterial community of the honeybees, and for each treatment, three replicates were undertaken. Pure sugar syrup (60% (w/v) sucrose solution) served as a negative control, and 48 ppb imidacloprid syrups were used as controls for exposure to a sublethal concentration of toxic product. The other three treatments were 20 ng/mL, 200 ng/mL, and 20 vμg/mL of Cry1Ie toxin syrups. In total, 2 mL of the sugar solution was provided per bee, and 2 g of fresh pollen-food per cage were supplied daily from a gravity feeder fitted on each cage^9^. The Cry1Ie toxin treatment continued for the entire experimental period, while, the pollen-food was only offered for the first 21 days^9^. Bees were collected for further analysis at 15 and 30 d.

### Honeybee dissection

The honeybees were dissected at 15 and 30 d after treatment. The bees were placed at −20 °C for 15 s before dissection, and five samples were randomly collected for each cage. The midguts of the honeybees were isolated in sterile conditions, kept in 2 mL eppendorf tubes and immediately stored at −80 °C until use.

### DNA extraction and quantitative PCR

Bacterial DNA from the midgut and pollen samples was extracted using the QIAamp DNA Stool Mini Kit (QIAGEN, Germany) according to the manufacturer’s instructions. The extracted bacterial DNA was evaluated by Eppendorf BioSpectrometer and stored at −20 °C before PCR analysis.

The abundance of the bacterial 16S ribosomal RNA (rRNA) gene was measured by real-time qPCR in an ABI7500 PCR System (Applied Biosystems, Carlsbad, CA) using the primer pairs 27F(5′-AGAGTTTGATCCTGGCTCAG-3′) and 355R(5′-CTGCTGCCTCCCGTAGGAGT-3′)^40^. To improve PCR efficiency, the touchdown procedure was conducted using the following conditions: 95 °C × 3 min for 1 cycle; 95 °C × 20 s, 66 °C × 10 s, for 5 cycles by decrease 1 °C per cycle, 68 °C × 20 s; followed by 35 cycles of 95 °C × 15 s, 60 °C × 15 s, 68 °C × 20 s. Each reaction was performed in a 20 μL volume containing 10 μL 2 × Es Taq, 0.5 μL of each primer (10 μM) and 1 μL of DNA template. All qPCR reactions for test samples were run in triplicate to avert technical error. Standard curves were constructed using serially diluted plasmid DNA containing the correct insert of the bacterial 16S rRNA gene. The copy number of the 16S rRNA gene was determined by linear regression of the normalized sample Ct values to the standard curve.

### MiSeq sequencing of 16S rRNA gene amplicons

16S rRNA gene amplicons were used to determine the diversity and structure comparisons of the bacterial species in each of these samples using Illumina MiSeq sequencing at Novogene Bioinformatics Technology Co., Ltd, Beijing, China. PCR amplifications were conducted with the barcoded primer pair 341f/806r set that amplifies the V3–V4 fragments of the 16S rDNA gene (341F: CCTAYGGGRBGCASCAG, 806: GGACTACNNGGGTATCTAAT)^41^,^42^. At first, all the initial template DNA concentrations were normalized between samples. PCR reactions were performed in a volume of 30 μL containing 12 μL sterile water, 1.0 μL DNA template, 1.0 μL of each primer, and 15 μL 2× Phusion Master Mix (New England Biolabs, USA). The PCR cycle conditions were as follows: initial denaturation at 98 °C for 1 min, followed by 30 cycles at 98 °C for 10 s, 50 °C for 30 s, and 72 °C for 30 s, and a final extension step at 72 °C for 5 min. The PCR products (approximately 200 bp) were separated by electrophoresis in agarose gels (2%, w/v), purified with Qiagen Gel Extraction Kit (Qiagen, Germany), and then pooled at equal concentrations. Pyrosequencing was conducted on an Illumina MiSeq 250 platform according to protocols described by Caporaso^42^.

### Data Analysis

Paired-end reads were assigned to each sample based on their unique barcode and truncated by cutting off the barcode and primer sequence. After initial trimming, we obtained the merged reads with FLASH v1.2.7 (http://ccb.jhu.edu/software/FLASH/) based on overlapping regions within paired-end reads^43^, and the splicing sequences were called raw tags. Qualities filtering on the raw tags were performed according to the QIIME (V1.7.0, http://qiime.org/index.html) quality control process^44^, and all sequences shorter than 2000 bp in length and a quality score lower than 25 in the raw reads were removed. The chimaera sequences were also removed through the UCHIME algorithm (UCHIME Algorithm, http://www.drive5.com/usearch/manual/uchime_algo.html)^45^.

Finally, we obtained the high-quality clean tags. These sequences were classified into the same OTUs (operational taxonomic units) at an identity threshold of 97% similarity using UPARSE software (UPARSE v7.0.1001, http://drive5.com/uparse/)^46^. For each OTU, a representative sequence was screened and used to assign taxonomic composition using the SILVA SSU database 119^47^,^48^. The taxon abundance of each sample was generated into phylum, class, order, family, and genera levels. All of the analyses from clustering to alpha (within sample) and beta diversity (between samples) were performed with QIIME (Version 1.7.0) and displayed with R software (Version 2.15.3).

### Statistical Analysis

Significant differences of the bacterial abundance across different treatments were analysed by performing one-way ANOVA (SPSS. 16.0) on the copy numbers of the 6S rRNA gene. The 16S rRNA gene copies were normalized with log transformation prior to statistical analysis. The richness estimators and the composition of the dominant midgut bacterial genera were also analysed by one-way ANOVA (SPSS. 16.0).

## Additional Information

**Accession codes:** Complete data sets for all samples have been deposited in the National Center for Biotechnology Information (NCBI) Sequence Reads Archive (SRA) under accession number SRP066948 (http://www.ncbi.nlm.nih.gov/Traces/sra/). 

**How to cite this article**: Jia, H.-R. *et al*. The effects of Bt Cry1Ie toxin on bacterial diversity in the midgut of *Apis mellifera ligustica* (Hymenoptera: Apidae). *Sci. Rep.* **6**, 24664; doi: 10.1038/srep24664 (2016).

## Supplementary Material

Supplementary Information
