# Supplementary material for: The effects of Bt Cry1Ie toxin on bacterial diversity in the midgut of Apis mellifera ligustica (Hymenoptera: Apidae)
Source: Sci Rep. 2016 Apr 19;6:24664. doi: 10.1038/srep24664 (PMC4835784; doi:10.1038/srep24664)

The effects of Bt Cry1Ie toxin on bacterial diversity in the midgut of *Apis mellifera ligustica* (Hymenoptera: Apidae)

Hui-Ru Jia[1, 2], Li-Li Geng[2], Yun-He Li[2], Qiang Wang[1], Qing-Yun Diao[1], Ting Zhou[1], Ping-Li Dai[1]*

1. Ministry Key Laboratory of Pollinating Insect Biology, Institute of Apicultural Research, Chinese Academy of Agricultural Sciences, Beijing 100093, China

2. State Key Laboratory for Biology of Plant Diseases and Insect Pests, Institute of Plant Protection, Chinese Academy of Agricultural Sciences, Beijing 100193, China

*Corresponding author. Tel. (fax):+86 10 62597285. E-mail address: daipingli@caas.cn.

Additional information

Table S1 16S rRNA gene copy numbers per sample.

Table S2 Statistical analysis of the copy number of 16S rRNA gene of each treatment by one-way ANOVA (SPSS. 16.0).

Table S3 OUT's data. Data was obtained using the Illumina Miseq2500-pyrosequencing.

Table S4 Statistical analysis of the composition of dominant midgut bacterial genera among different groups by one-way ANOVA (SPSS. 16.0).

Table S5 Coverage, Shannon, chao1 and ACE richness estimator.

Table S6 Statistical analysis of richness estimator among different groups by one-way ANOVA (SPSS. 16.0).

Table S7 ANOSIM statistical analysis among different groups at 2 sampling time, respectively.

Figure S1 Standard curve of qPCR.

Figure S2 Rarefaction on species-abundance data. Average value of 3 replicates were showed.

Figure S3 OTU abundances and taxonomic classifications within each sample at different levels.

Supplemental Data

Table S1 16S rRNA gene copy numbers per sample.

Sample	ct1	ct2	ct3	LOG10 (Copies 1）	LOG10 (Copies 2）	LOG10 (Copies 3）	Copes 1 (copies/μl)	Copes 2 (copies/μl)	Copes 3 (copies/μl)
CK-A1	13.06	12.28	13.32	5.580832	5.863816	5.486504	3.81E+05	7.31E+05	3.07E+05
CK-A2	13.52	13.54	13.56	5.413944	5.406688	5.399432	2.59E+05	2.55E+05	2.51E+05
CK-A3	12.46	12.06	12.95	5.798512	5.943632	5.62074	6.29E+05	8.78E+05	4.18E+05
T1-A1	15.9	16.3	16.5	4.55048	4.40536	4.3328	3.55E+04	2.54E+04	2.15E+04
T1-A2	15.69	15.8	16.13	4.626668	4.58676	4.467036	4.23E+04	3.86E+04	2.93E+04
T1-A3	14.67	14.68	14.01	4.996724	4.993096	5.236172	9.92E+04	9.84E+04	1.72E+05
T2-A1	11.98	11.99	11.86	5.972656	5.969028	6.016192	9.39E+05	9.31E+05	1.04E+06
T2-A2	13.89	14.11	14.43	5.279708	5.199892	5.083796	1.90E+05	1.58E+05	1.21E+05
T2-A3	14.86	14.85	14.64	4.927792	4.93142	5.007608	8.47E+04	8.54E+04	1.02E+05
T3-A1	14.58	14.9	15.16	5.029376	4.91328	4.818952	1.07E+05	8.19E+04	6.59E+04
T3-A2	13.52	13.33	13.9	5.413944	5.482876	5.27608	2.59E+05	3.04E+05	1.89E+05
T3-A3	12.68	12.82	12.96	5.718696	5.667904	5.617112	5.23E+05	4.65E+05	4.14E+05
IMI-A1	14.21	14.67	14.53	5.163612	4.996724	5.047516	1.46E+05	9.92E+04	1.12E+05
IMI-A2	14.85	15.57	15.34	4.93142	4.670204	4.753648	8.54E+04	4.68E+04	5.67E+04
IMI-A3	14.02	13.73	13.77	5.232544	5.337756	5.323244	1.71E+05	2.18E+05	2.10E+05
CK-B1	14.58	14.31	14.52	5.029376	5.127332	5.051144	1.07E+05	1.34E+05	1.12E+05
CK-B2	14.67	14.58	14.81	4.996724	5.029376	4.945932	9.92E+04	1.07E+05	8.83E+04
CK-B3	13.96	13.87	13.72	5.254312	5.286964	5.341384	1.80E+05	1.94E+05	2.19E+05
T1-B1	14.36	14.33	14.94	5.109192	5.120076	4.898768	1.29E+05	1.32E+05	7.92E+04
T1-B2	13.56	13.93	13.64	5.399432	5.265196	5.370408	2.51E+05	1.84E+05	2.35E+05
T1-B3	14.67	14.13	14.09	4.996724	5.192636	5.207148	9.92E+04	1.56E+05	1.61E+05
T2-B1	14.06	14.6	14.52	5.218032	5.02212	5.051144	1.65E+05	1.05E+05	1.12E+05
T2-B2	15.47	14.91	15.21	4.706484	4.909652	4.800812	5.09E+04	8.12E+04	6.32E+04
T2-B3	11.92	12.2	11.77	5.994424	5.89284	6.048844	9.87E+05	7.81E+05	1.12E+06
T3-B1	12.98	12.66	12.76	5.609856	5.725952	5.689672	4.07E+05	5.32E+05	4.89E+05
T3-B2	12.68	12.86	12.64	5.718696	5.653392	5.733208	5.23E+05	4.50E+05	5.41E+05
T3-B3	12.94	13.16	12.7	5.624368	5.544552	5.71144	4.21E+05	3.50E+05	5.15E+05
IMI-B1	12.44	12.69	13.16	5.805768	5.715068	5.544552	6.39E+05	5.19E+05	3.50E+05
IMI-B2	13	12.74	12.57	5.6026	5.696928	5.758604	4.00E+05	4.98E+05	5.74E+05
IMI-B3	12.68	12.71	12.91	5.718696	5.707812	5.635252	5.23E+05	5.10E+05	4.32E+05

Table S2 Statistical analysis of the copy number of 16S rRNA gene of each treatment by one-way ANOVA (SPSS. 16.0).

groups	F	df	*P*
CK.15d, T1.15d ,T2.15d, T3.15d, IMI.15d	2.312	14	0.129
CK.30d, T1.30d ,T2.30d, T3.30d, IMI.30d	2.619	14	0.099

Table S3 OUT's data. Data was obtained using the Illumina Miseq2500-pyrosequencing.

Sample Name	Raw PE	Combined	Qualified	Base(nt)	Avg Len(nt)	Effective%
Ck.A.1	47,147	45,008	40,900	16,928,062	417	86.11
Ck.A.2	40,398	38,500	34,773	14,366,490	417	85.18
Ck.A.3	52,726	49,971	44,746	18,817,783	423	84.35
Ck.B.1	47,659	45,269	41,051	16,850,765	414	85.48
Ck.B.2	42,651	40,524	36,776	15,148,549	414	85.73
Ck.B.3	53,909	51,368	46,561	19,136,620	414	85.73
IMI.A.1	49,754	46,962	42,094	17,562,379	421	83.91
IMI.A.2	53,125	50,300	45,070	18,948,701	424	84.16
IMI.A.3	57,764	54,909	49,827	20,565,816	415	85.69
IMI.B.1	51,373	48,666	43,590	18,213,876	421	84.21
IMI.B.2	42,382	40,186	35,646	15,025,778	425	83.49
IMI.B.3	54,404	51,897	46,748	19,493,870	420	85.35
T1.A.1	57,016	53,523	47,220	19,985,691	427	82.15
T1.A.2	58,692	55,627	50,625	20,718,205	414	85.3
T1.A.3	42,332	39,987	35,766	14,757,078	422	82.56
T1.B.1	40,728	38,489	34,294	14,112,296	424	81.81
T1.B.2	56,966	54,528	49,737	20,272,867	412	86.4
T1.B.3	49,519	47,047	42,349	17,445,691	417	84.53
T2.A.1	58,036	55,298	50,321	20,441,251	412	85.55
T2.A.2	55,724	53,060	48,163	19,624,564	415	84.96
T2.A.3	48,532	45,904	41,227	17,085,783	419	84.09
T2.B.1	45,157	43,061	39,175	16,014,857	412	86.06
T2.B.2	50,199	47,722	43,075	17,809,600	417	84.99
T2.B.3	54,439	51,959	47,380	19,408,794	413	86.37
T3.A.1	53,403	50,025	44,430	18,405,536	420	82.12
T3.A.2	42,621	40,247	35,807	15,103,236	426	83.27
T3.A.3	40,579	38,597	35,017	14,362,396	414	85.57
T3.B.1	58,224	55,281	49,844	20,660,652	419	84.6
T3.B.2	46,862	44,669	40,462	16,833,919	419	85.77
T3.B.3	58,917	55,932	50,480	20,894,710	417	84.95

Table S4 Statistical analysis of the composition of dominant midgut bacterial genera among different groups by one-way ANOVA (SPSS. 16.0).

genera	groups	F	df	*P*
Gilliamella	CK.15d, T1.15d ,T2.15d, T3.15d, IMI.15d	0.771	14	0.568
	CK.30d, T1.30d ,T2.30d, T3.30d, IMI.30d	1.690	14	0.228
Frischella	CK.15d, T1.15d ,T2.15d, T3.15d, IMI.15d	0.214	14	0.925
	CK.30d, T1.30d ,T2.30d, T3.30d, IMI.30d	0.361	14	0.831
Snodgrassella	CK.15d, T1.15d ,T2.15d, T3.15d, IMI.15d	3.397	14	0.053
	CK.30d, T1.30d ,T2.30d, T3.30d, IMI.30d	4.244	14	0.059
Enterobacter	CK.15d, T1.15d ,T2.15d, T3.15d, IMI.15d	3.865	14	0.058
	CK.30d, T1.30d ,T2.30d, T3.30d, IMI.30d	1.320	14	0.327
Bifidobacterium	CK.15d, T1.15d ,T2.15d, T3.15d, IMI.15d	4.242	14	0.069
	CK.30d, T1.30d ,T2.30d, T3.30d, IMI.30d	1.245	14	0.353
Lactobacillus	CK.15d, T1.15d ,T2.15d, T3.15d, IMI.15d	2.219	14	0.140
	CK.30d, T1.30d ,T2.30d, T3.30d, IMI.30d	1.063	14	0.424
Citrobacter	CK.15d, T1.15d ,T2.15d, T3.15d, IMI.15d	0.858	14	0.521
	CK.30d, T1.30d ,T2.30d, T3.30d, IMI.30d	0.846	14	0.527
Commensalibacter	CK.15d, T1.15d ,T2.15d, T3.15d, IMI.15d	2.093	14	0.157
	CK.30d, T1.30d ,T2.30d, T3.30d, IMI.30d	4.884	14	0.089
Saccharibacter	CK.15d, T1.15d ,T2.15d, T3.15d, IMI.15d	1.808	14	0.204
	CK.30d, T1.30d ,T2.30d, T3.30d, IMI.30d	0.608	14	0.666
Others	CK.15d, T1.15d ,T2.15d, T3.15d, IMI.15d	2.129	14	0.152
	CK.30d, T1.30d ,T2.30d, T3.30d, IMI.30d	5.121	14	0.067

Table S5 richness estimator of 30 samples

Sample name	observed_ species	Shannon	Simpson	chao1	ACE	goods_ coverage
Ck.15d1	73	2.477	0.745	80	84.749	1
Ck.15d2	83	2.788	0.801	92	100.705	0.999
Ck.15d3	103	2.255	0.648	117.882	122.016	0.999
Ck.30d1	64	2.974	0.813	69.353	74.389	1
Ck.30d2	36	2.449	0.743	54.333	54.4	1
Ck.30d3	72	2.304	0.674	75.391	81.036	1
T1.15d1	108	2.528	0.74	114.364	125.903	0.999
T1.15d2	142	2.404	0.655	215.929	185.883	0.999
T1.15d3	172	3.417	0.851	182	181.332	0.999
T1.30d1	163	3.159	0.808	308.462	245.611	0.998
T1.30d2	89	1.92	0.537	97.55	105.052	0.999
T1.30d3	82	2.725	0.778	103.429	107.113	0.999
T2.15d1	120	2.338	0.597	140.3	144.946	0.999
T2.15d2	109	2.7	0.723	122.542	133.606	0.999
T2.15d3	116	2.673	0.764	134.4	133.021	0.999
T2.30d1	110	2.815	0.765	131	133.964	0.999
T2.30d2	103	2.982	0.824	116.316	120.054	0.999
T2.30d3	127	2.484	0.691	141.512	154.24	0.999
T3.15d1	121	3.079	0.837	143.895	149.145	0.999
T3.15d2	86	2.394	0.73	93.5	95.365	1
T3.15d3	66	2.589	0.734	75.1	75.748	1
T3.30d1	66	3.031	0.852	77.053	88.639	0.999
T3.30d2	78	2.396	0.733	96.071	105.248	0.999
T3.30d3	46	2.466	0.755	68.667	74.01	0.999
IMI.15d1	54	2.441	0.774	63.1	65.988	1
IMI.15d2	54	2.419	0.77	61.8	69.43	1
IMI.15d3	44	2.461	0.733	49	56.251	1
IMI.30d1	36	2.826	0.829	38.625	40.815	1
IMI.30d2	48	3.027	0.818	52	53.88	1
IMI.30d3	61	2.667	0.796	70.714	82.959	0.999

Table S6 Statistical analysis of Shannon index using SPSS 16.0.

groups	F	df	*P*
CK.15d, T1.15d ,T2.15d, T3.15d, IMI.15d	0.526	14	0.719
CK.30d, T1.30d ,T2.30d, T3.30d, IMI.30d	0.262	14	0.896

Table S7 ANOSIM statistical analysis among different groups at 2 sampling time, respectively.

groups	F	*P*
T1.15d-Ck.15d	-0.1111	0.7
T1.15d-T2.15d	0.1481	0.4
T1.15d-T3.15d	-0.2222	0.7
T1.15d-IMI.15d	-0.1852	0.8
T2.15d-Ck.15d	0.1852	0.2
T2.15d-T3.15d	0.1481	0.2
T2.15d-IMI.15d	0.3704	0.2
T3.15d-Ck.15d	-0.1852	0.7
T3.15d-IMI.15d	-0.1852	0.7
IMI.15d-Ck.15d	-0.1852	0.8
T1.30d-Ck.30d	-0.1111	0.8
T1.30d-T2.30d	0.03704	0.5
T1.30d-T3.30d	-0.1481	0.9
T1.30d-IMI.30d	0.03704	0.5
T2.30d-Ck.30d	-0.2963	1
T2.30d-T3.30d	0.1481	0.3
T2.30d-IMI.30d	0.4444	0.1
T3.30d-Ck.30d	0.03704	0.4
T3.30d-IMI.30d	-0.07407	0.7
IMI.30d-Ck.30d	0.3704	0.1

Figure S1 Standard curve of qPCR.

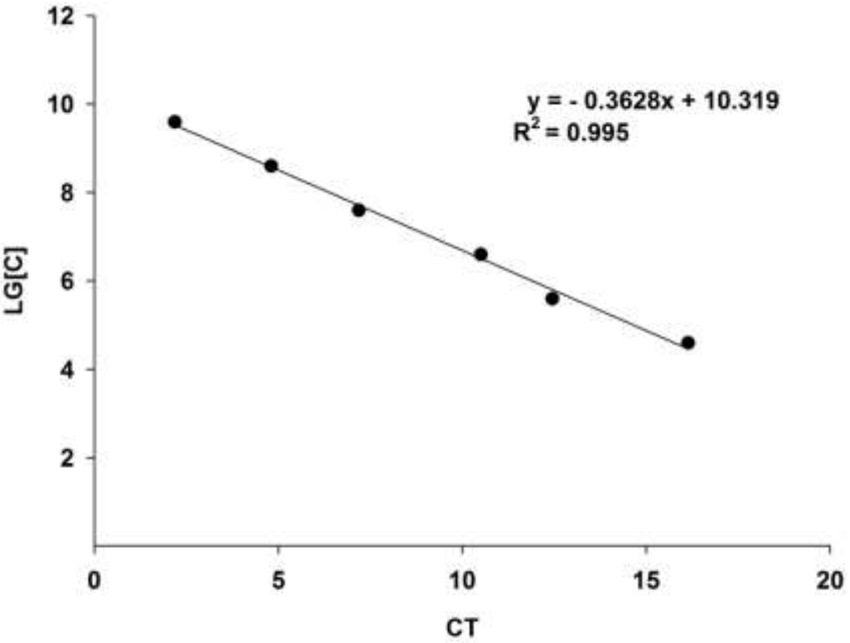

Figure S2 Rarefaction on species-abundance data. Average value of 3 replicates were showed.

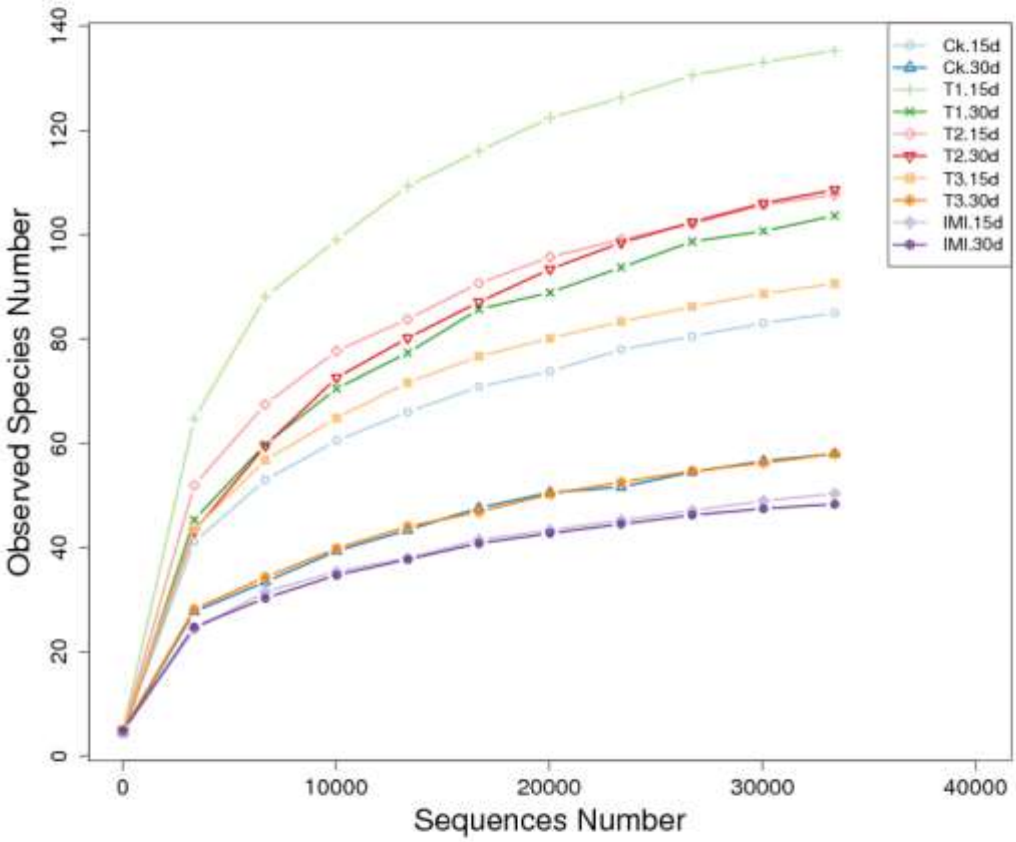

Figure S3 OTU abundances and taxonomic classifications within each sample at different levels.

Phylum

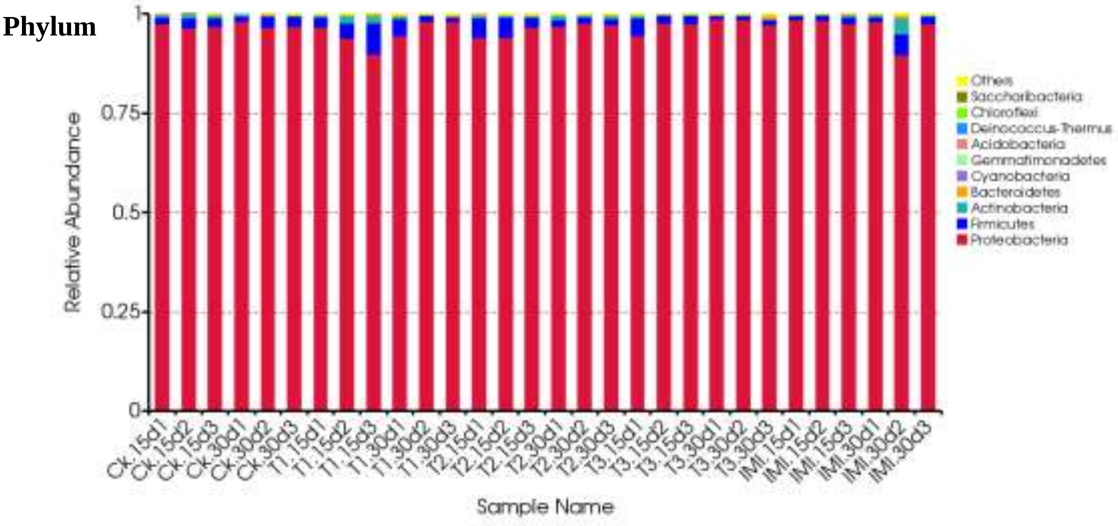

Class

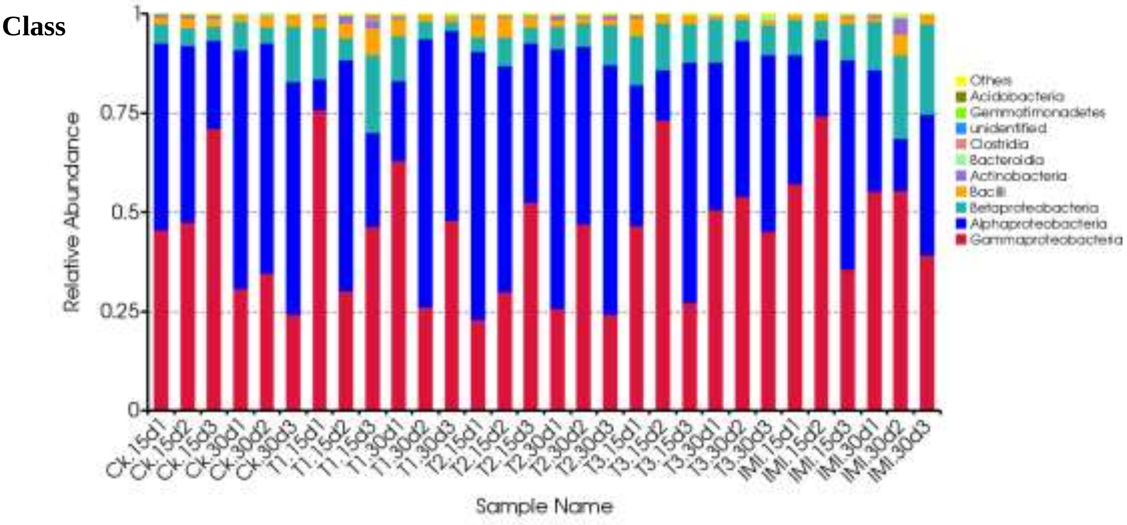

Order

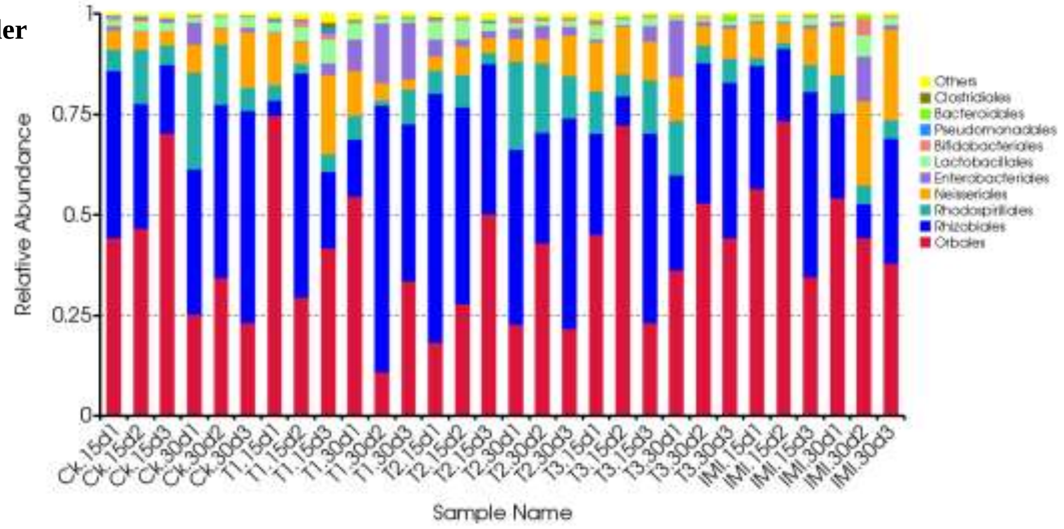

Family

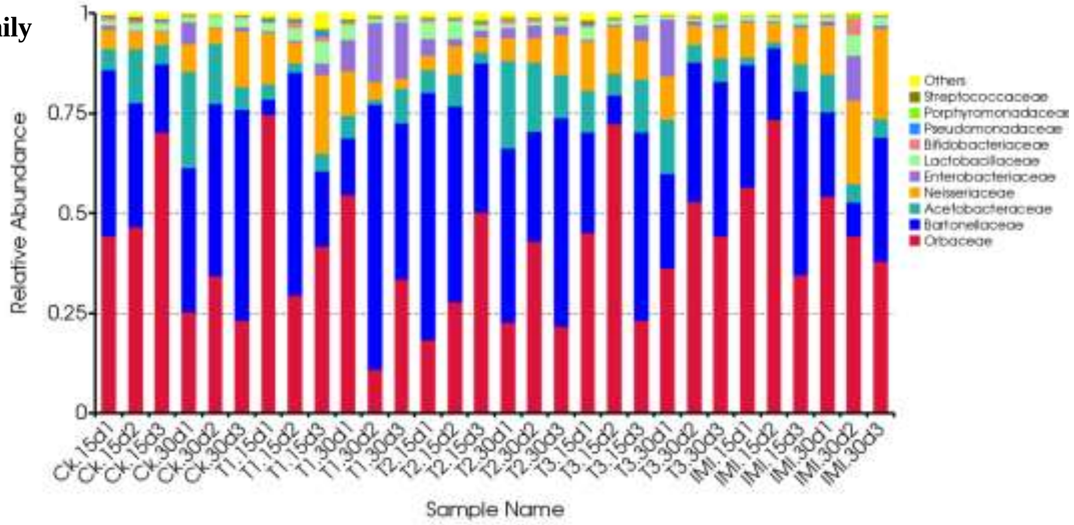

Supplement: Supplementary Information [file srep24664-s1.pdf]
